# A Comparative Review of Biological, Electrochemical, and Membrane-Based Methods for Direct Ocean Carbon Capture

**DOI:** 10.3390/ma19091763

**Published:** 2026-04-26

**Authors:** Zhe Wang, Jiayu Zheng, Siyuan Guo, Ting Zhang, Zhen Wang, Hang Cao, Gang Kevin Li, Shupeng Li, Yi Yang

**Affiliations:** 1 College of Ocean Engineering and Energy, Guangdong Ocean University, Zhanjiang 524088, China; wz0505@gdou.edu.cn (Z.W.); 13580430730@163.com (J.Z.); 18320930661@163.com (S.G.); 13085774496@163.com (T.Z.); 2 School of Mechanical Engineering, Xi’an Jiaotong University, Xi’an 710049, China; 2234112355@stu.xjtu.edu.cn; 3 State Environmental Protection Key Laboratory of Eco-Industry, Northeastern University, Shenyang 110819, China; caohang@mails.neu.edu.cn; 4 Department of Chemical Engineering, The University of Melbourne, Parkville, VIC 3010, Australia; li.g@unimelb.edu.au

**Keywords:** direct ocean carbon capture, electrochemical carbon capture, microalgae, hollow fiber membrane

## Abstract

Direct ocean carbon capture (DOC) has emerged as a promising strategy for mitigating atmospheric CO_2_ levels and addressing ocean acidification. Unlike direct air carbon capture methods, DOC leverages the ocean’s vast carbon storage capacity, offering a scalable and efficient route for carbon dioxide removal. This systematic comparative review categorizes existing DOC methods into three types: (1) biological carbon capture, which relies on photosynthesis by microalgae and marine microorganisms; (2) electrochemical carbon capture, which utilizes water electrolysis to generate H^+^ and OH^−^ ions for pH-driven CO_2_ removal; and (3) physical carbon capture, which employs hollow fiber membranes to directly separate CO_2_ from seawater. For each technology, we evaluate efficiency, energy consumption, cost, technology readiness level (TRL), scalability, and major challenges. By integrating recent pilot data and providing a critical assessment, this review offers a roadmap for future research in direct seawater CO_2_ capture. The comparative analysis reveals that electrochemical methods achieve the highest efficiency (60–85%) but face membrane fouling and electrode degradation challenges, while biological methods offer low-energy operation but suffer from slow kinetics and high harvesting costs, and membrane-based methods provide high removal rates (up to 94%) but require improved fouling resistance.

## 1. Introduction

Among the numerous severe challenges facing the world today, carbon dioxide (CO_2_) emissions have emerged as a central driver of climate change, exerting far-reaching and complex impacts on global ecosystems [1]. According to the Intergovernmental Panel on Climate Change (IPCC) [2], the continued accumulation of atmospheric CO_2_ will exacerbate global warming [3]. While the ocean’s natural capacity for CO_2_ absorption is vast, the rate of passive uptake is insufficient to meet the urgent requirements of global decarbonization [4,5,6,7]. Consequently, engineered solutions such as direct ocean capture (DOC) have gained prominence for their potential to actively accelerate carbon removal and directly mitigate localized ocean acidification [4,5,6,7]. However, the transition from theoretical feasibility to industrial-scale deployment is impeded by the formidable complexity of the marine environment [8]. Unlike land-based systems, DOC technologies must maintain high capture efficiency while enduring extreme conditions, including hydrostatic pressure in deep-sea regions, the highly corrosive nature of seawater, and operational disruptions caused by variable ocean currents and biofouling [4,5,6,7].

Covering approximately 71% of Earth’s surface, the ocean plays an indispensable role in the global carbon cycle [9]. Since the onset of the Industrial Revolution, the ocean has absorbed nearly 40% of anthropogenic CO_2_ emissions [10,11]. However, this uptake has significantly altered seawater chemistry, leading to ocean acidification with profound negative consequences for marine ecosystems [12]. For instance, many marine organisms like corals and shellfish have shells and skeletons mainly composed of calcium carbonate [13]. The increased acidity can dissolve calcium carbonate, affecting the growth, development, and survival of these organisms [14,15,16,17]. By reducing CO_2_ emissions into the atmosphere, carbon capture technologies can alleviate the pressure on oceanic CO_2_ uptake and help mitigate further acidification, offering a viable pathway for marine ecosystem protection [18].

**Figure 1:**
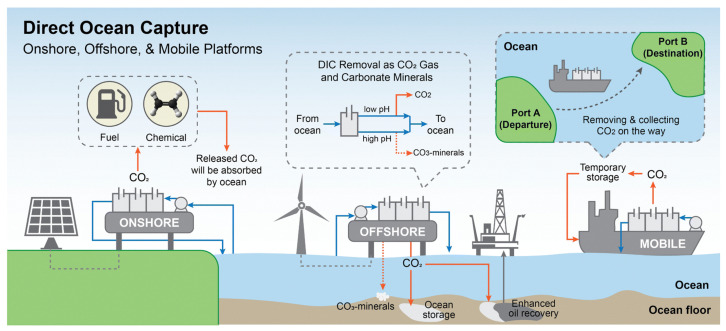
Three forms of deployment for direct ocean capture: onshore, offshore and mobile [20]. Reprinted with permission from [20]. Copyright 2023 Royal Society of Chemistry.

At present, carbon capture technologies are multiplying and have been a popular research topic. Among them, direct air carbon capture (DAC) and direct ocean carbon capture (DOC) have drawn particular attention, and many researchers have conducted in-depth explorations of the practical application of these two technologies in decarbonization strategies [19]. For instance, recent pilot projects have demonstrated that DOC can effectively reduce CO_2_ concentrations in localized marine areas by leveraging natural carbon cycling mechanisms (Figure 1) [20]. This not only opens up a new path for carbon sequestration but also holds promise for alleviating the severe problem of ocean acidification [21]. Despite these advances, DOC technology remains immature and faces significant technical and environmental challenges [22]. From a technical perspective, the complexity and harshness of the marine environment impose extremely stringent requirements on the durability and efficiency of carbon capture equipment [23]. High-pressure conditions in deep-sea regions, the highly corrosive nature of seawater, and complex, variable ocean currents can lead to equipment corrosion, blockage, and operational failures, thereby limiting large-scale deployment [24,25,26]. Environmentally, ocean-based carbon capture methods may trigger cascading effects within marine ecosystems, necessitating careful assessment [27].

While the existing literature offers extensive insights into individual carbon capture methods, this review provides a unique contribution by systematically integrating biological, electrochemical, and physical approaches into a unified comparative framework. Specifically, we examine microalgae-based carbon capture (with an emphasis on cyanobacteria) in biological methods, electrochemical capture based on water electrolysis, and hollow fiber membrane-based physical capture. We analyze the fundamental principles and relevant data for each approach, evaluate their feasibility, and summarize the latest research findings to serve as a reference for future studies.

## 2. Review Methodology

This review follows a systematic comparative approach to evaluate direct ocean carbon capture (DOC) technologies. The literature search was conducted using three databases: Web of Science, Scopus, and Google Scholar, covering the period from 2012 to 2025. The search keywords included: “direct ocean carbon capture”, “marine carbon capture”, “electrochemical carbon capture”, “bipolar membrane electrodialysis seawater”, “microalgae carbon capture”, and “hollow fiber membrane CO_2_ seawater”. Only peer-reviewed journal articles and authoritative reports (e.g., IPCC, IEA) written in English were included. Studies were selected as important references if they reported quantitative performance metrics (e.g., CO_2_ capture efficiency, energy consumption, cost estimates) or provided substantive technological descriptions relevant to DOC. The review is comparative in nature, applying consistent evaluation criteria—including efficiency, energy consumption, cost, TRL, scalability, and major challenges—across all three technology categories (biological, electrochemical, and membrane-based). Data extraction focused on these metrics to enable direct cross-technology comparison.

## 3. Overview of DOC Technologies

Biological carbon capture: The oceans are the largest natural sink for carbon dioxide and are thought to have absorbed at least 2.5 peta-grams of carbon per year (PgC yr^−1^) of anthropogenic carbon from 1994 to 2007 [28]. Marine ecosystems comprise a wide variety of life forms, most of which are unicellular archaea, bacteria, and eukaryotes. The ability of these microorganisms to process carbon, shape Earth’s atmosphere, and fuel the ocean food web has been well demonstrated [29]. Carbon in the oceans can also be utilized by aquatic organisms such as marine organisms, corals and shellfish. The traditional use of microalgae in seawater is the main means of biological carbon capture [30]. The production economics of the microalgae pathway are complex, with the estimated CO_2_ utilization potential of microalgae by 2050 ranging from 0.2 to 0.9 Gt CO_2_/year, and with breakeven cost quartiles ranging from $230 to $920 per ton of CO_2_ in the range reviewed [31].

Electrochemical carbon capture: Electrochemical carbon capture has a long history of development, mainly through direct or indirect electrolyte hydrolysis of seawater flowing through the electrodes of the water reaction to produce H^+^ and OH^−^ ions [32]. Among them, H^+^ can create an acidic environment that allows seawater to produce CO_2_ in the HCO_3_^−^ decomposition reaction for subsequent capture, and the OH^−^ creates an alkaline environment to generate CO_3_^2−^ followed by the formation of insoluble carbonate precipitates for recovery [20]. DOC technology using electrochemical methods is dominated by bipolar membrane electrodialysis (BPMED); it also includes other technologies such as electrochemical hydrogen-looping (EHL), monopole membrane electrodialysis, and an asymmetric chloride-mediated electrochemical process [33]. BPMED technology consists of a membrane unit with different cation/anion exchange membranes set at regular intervals and electrode solutions filled on both sides, where seawater serves as the raw material for the hydrolysis reaction [34,35]. The subsequent decarbonization of seawater is achieved by stripping CO_2_ from acidified solutions using membrane contactors and separating carbonates from alkaline solutions using filtration or by treating the precipitates in a plant [36,37]. The EHL technology, on the other hand, is based on three-cell work, and utilizes the hydrogen generated from the hydrolysis reaction to re-electrolyze to generate H^+^ in order to further reduce the pH of the acidified solution, and it realizes low-pressure electrochemical CO_2_ extraction by taking advantage of the small potential difference between hydrogen oxidation and evolution (HOR/HER) in acidic and alkaline solutions, respectively [38]. Monopole membrane electrodialysis is a technique that uses an anion exchange membrane (AEM) or cation exchange membrane (CEM) to electrolyze a solution that can generate an acidic or basic stream to capture CO_2_ [39]. Direct seawater electrolysis facilitates CO_2_ capture by creating an alkaline environment through the production of OH^−^ after direct electrolysis of seawater by different electrodes, and an asymmetric chloride-mediated electrochemical process is employed to capture carbon through a one-way device with a changeable direction [40]. As an emerging method of marine carbon capture, electrochemical carbon capture still has significant potential for multi-directional development in the future [41].

Physical carbon capture: This review specifically focuses on membrane-based contactors for DOC applications. Hollow fiber membrane technology is particularly reviewed in this study due to its popularity. As an efficient membrane separation technology, the hollow fiber membrane plays a vital role in many fields like modern industry and environmental science, demonstrating unique advantages through its distinctive structure and properties [42]. The hollow fiber membrane is made of polymer materials with a specific pore structure and chemical properties, featuring a hollow fiber-like structure that varies in fiber diameter from tens to hundreds of microns [43]. It has a large specific surface area, which provides a wide range of membrane separation area per unit volume to improve the separation efficiency [44]. The hollow fiber membrane was initially used in the field of artificial kidneys (hemodialysis) for key substance exchange functions [45,46]. Subsequently, with the continuous development of technology, its application has gradually expanded to various areas such as gas separation, sewage purification, and seawater desalination [47,48]. Given the increasingly mature substance separation technology of the hollow fiber membrane, its application potential in ocean carbon capture has drawn much attention [49]. The working principle of the hollow fiber membrane in the DOC process mainly involves adsorption, diffusion, selective permeation, and enrichment collection [50]. Special chemical groups or physical structures on membrane surfaces adsorb CO_2_ from seawater. The concentration difference between both sides prompts CO_2_ to diffuse across the membrane, which allows CO_2_ to pass through preferentially while blocking other components in seawater [51]. The CO_2_ that passes through is enriched and collected on the other side of the membrane and removed from the marine environment after subsequent treatment, thus realizing ocean carbon capture [52].

Comparative Summary of DOC Pathways: While biological, electrochemical, and physical methods all aim to extract CO_2_ from the marine environment, they differ significantly in their fundamental mechanisms and operational requirements. Biological methods leverage natural metabolic processes but are often limited by slow kinetics and high area requirements. In contrast, electrochemical and membrane-based technologies offer higher capture intensities and smaller footprints, though they demand higher energy inputs and face material durability challenges in corrosive seawater. In addition, as illustrated in Figure 1, DOC technology offers spatial versatility across onshore, offshore, and mobile platforms, yet each configuration presents distinct trade-offs between applicability and operational constraints. Onshore systems leverage existing coastal facilities to reduce costs, whereas offshore platforms utilize vast marine spaces and integrated renewable energy but must withstand extreme hydrostatic pressure and severe seawater corrosion. Mobile configurations allow for CO_2_ capture during maritime transit but are currently constrained by high operational energy demands and complex carbon sequestration logistics. Overall, these platforms remain largely at the demonstration stage (TRL 4–6), with their commercial viability contingent upon overcoming material durability and economic scaling hurdles.

## 4. Progress of Biological Carbon Capture

**Figure 2:**
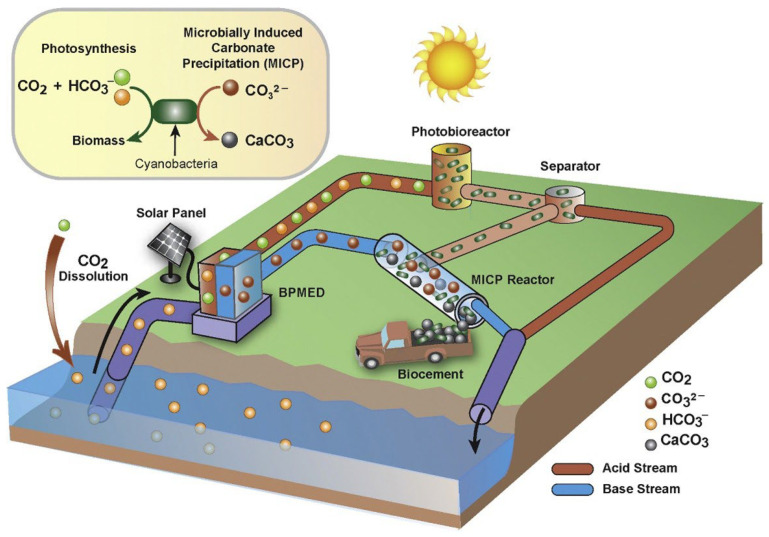
Schematic illustration of the proposed microbially induced carbonate precipitation (MICP) process [53]. Reprinted with permission from [53]. Copyright 2022 Elsevier.

Kludze et al. [53] proposed a hybrid approach that combines primary CO_2_ capture via autotrophic microbial growth (i.e., photosynthetic cyanobacteria) and microbially induced carbonate precipitation (MICP) (Figure 2). It is not only limited to carbon fixation through photosynthesis of cyanobacteria, but also incorporates the use of microbial excreta to facilitate the conversion of dissolved CO_2_ in seawater to CaCO_3_ precipitation, thus combining the natural carbon fixation of the former with the efficacy and persistence of the latter, as well as ameliorating seawater acidification to a certain extent. Compared to some conventional methods such as extraction via membrane contactors and vacuum pumps, this method does not require further conversion or mineralization of CO_2_, saving additional energy costs and carbon intensity [53].

However, although MICP represents one of the major research directions for biologically induced DOC, it still has deficiencies in terms of sustainability: urea hydrolysis re-leases ammonia as a byproduct, which poses a high risk of ecological pollution in aquatic environments [54]. To address these issues, the microalgae-induced carbonate precipitation (MAICP) proposed in recent research represents an entirely novel photosynthetic carbonate carbon sequestration pathway. It perfectly overcomes the drawbacks of conventional MICP while precisely matching the technical requirements of biological DOC [55]. Through photosynthesis, microalgae such as *Chlorella vulgaris* assimilate dissolved DIC from seawater, releasing OH^−^ ions that raise the ambient pH to an alkaline range of 9.5–9.8, thereby shifting the carbonate equilibrium toward CaCO_3_ supersaturation. Empirical validation demonstrated that, under optimized conditions, the system attained a CO_2_ fixation rate of 97.23 mg/L/d, a maximum total carbon capture capacity of 1943.37 mg/L, and an 87.43% calcium removal rate. Unlike conventional MICP, MAICP avoids ammonia byproducts and urea feedstock, offering superior marine adaptability. This process enhances the natural biological pump, providing an active and ecologically compatible solution for engineered marine carbon sequestration [55].

**Figure 3:**
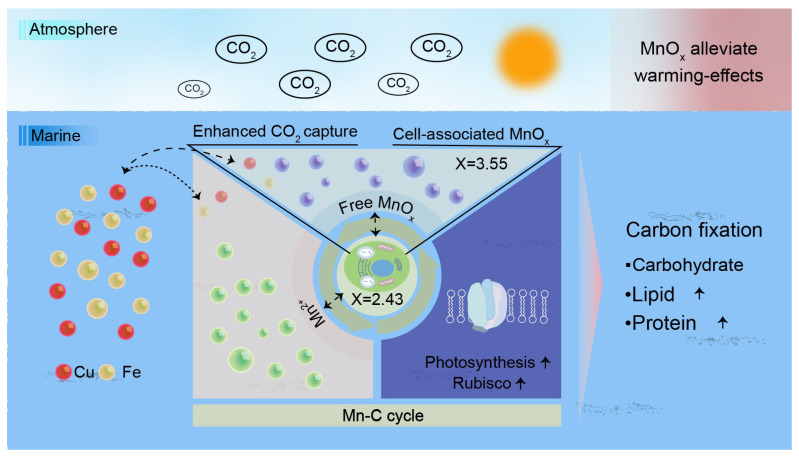
Manganese ions form self-assembled nanoscale MnOx in phytoplankton and increase biological carbon fixation [57].

Among engineered biological DOC technologies, the cultivation of microalgae and macroalgae has been most extensively studied to enhance carbon fixation efficiency by optimizing the growth conditions of photosynthetic organisms. You et al. [56] similarly concluded that microalgae are capable of fixing CO_2_ and utilizing nutrients from wastewater through photosynthesis, making them the most promising biotechnology for carbon capture. A method to introduce micro-nano-bubbles (MNBs) into the culture of microalgae has been proposed, as MNBs have the advantages of small size, high gas–liquid mass transfer efficiency, and high electrostatic interactions, showing the potential to improve CO_2_ solubility and the carbon capture efficiency of microalgae [56]. However, most existing studies are limited to laboratory-scale tests, and scaling up microalgae-based DOC faces challenges including slow growth kinetics (6–48 h doubling time), high harvesting costs (20–30% of production), large land and water footprints, environmental sensitivity, and contamination risks. These constraints, combined with high costs ($230–920/ton CO_2_), suggest that biological DOC is better suited for niche applications than large-scale rapid carbon removal. Hou et al. [57] demonstrated that self-assembled manganese oxides (MnOx) enhance diatom photosynthesis and CO_2_ capture by mimicking the Mn_4_CaO_5_ cluster in photosystem II, achieving a 1.5-fold increase in capture capacity through biohybrid formation (Figure 3). Compared to the MICP approach proposed by Kludze et al. [53], which achieves permanent mineral storage but requires careful microbial community management, and the MNBs method proposed by You et al. [56], which improves gas–liquid mass transfer but demands continuous energy input, the diatom/MnOx system offers a passive enhancement strategy with a higher reported enhancement factor. However, concerns over manganese release and long-term biohybrid stability, together with the lack of pilot-scale validation, currently limit its practical applicability for DOC. Li et al. [58] developed an electro-biocatalytic hybrid system that converts dissolved inorganic carbon from seawater into bioplastic monomers. They engineered Vibrio natriegens by introducing the Ftl-Fch-Mtd formate-assimilation pathway, enabling it to utilize formate as the sole carbon source for succinic acid production. Under shake-flask cultivation, the system yielded 1.13 g L^−1^ succinic acid. Scale-up to 1 L and 5 L industrial fermenters further improved titers to 1.16 g L^−1^ and 1.37 g L^−1^, respectively, with lactic acid produced as a byproduct at 0.25 g L^−1^. High-quality plastic poly(butylene succinate) bioplastic synthesized from the biosourced succinic acid demonstrated identical properties to petroleum-based poly(butylene succinate) [58].

Collectively, microalgal carbon capture is technically viable, yet economic feasibility remains the primary barrier to its practical deployment, necessitating full-chain optimization: novel cultivation systems to optimize light and CO_2_ distribution; wastewater-derived nutrient supply to couple carbon removal with wastewater treatment and cost reduction; strain engineering to enhance carbon fixation, stress tolerance, and high-value co-production; and optimized harvesting and water recycling [59]. Such integrated efforts are critical to enabling the large-scale implementation of this technology [60]. The natural ocean biological pump transports carbon fixed in the surface ocean to the deep sea for long-term sequestration via natural mechanisms, including gravitational sedimentation, biological vertical migration, and physical mixing [61,62], which serves as the core theoretical foundation for engineered biological DOC technologies.

## 5. Progress of Electrochemical Carbon Capture Technologies

**Figure 4:**
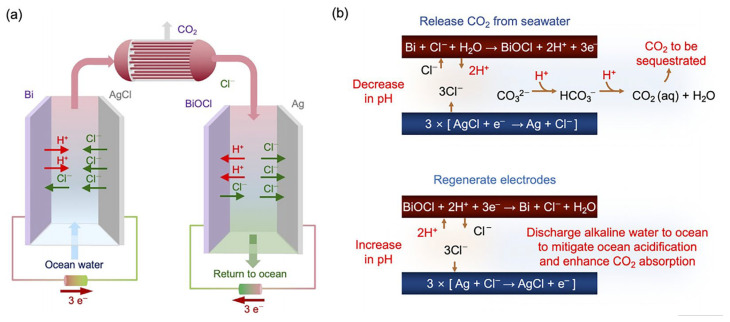
(**a**) General principle of the chloride-mediated electrochemical pH swing system for CO_2_ removal from ocean water; (**b**) electrochemical reactions at the bismuth (red) electrode and silver (blue) electrode in each step, and subsequent CO_2_ release in the acidified oceanwater [64]. Reprinted with permission from [64]. Copyright 2023 Royal Society of Chemistry.

Recent advances in electrochemical DOC have produced many distinct approaches with varying trade-offs between efficiency, energy consumption, and operational complexity [62]. Han et al. [63] developed a dual-chamber electrolysis model achieving complete calcium removal as CaCO_3_, but with relatively high energy consumption (558–730 kJ/mol CaCO_3_). Kim et al. [64]. proposed an asymmetric chloride-mediated process that eliminates ion-exchange membranes entirely, achieving 87% DIC removal, 92% Faraday efficiency, and significantly lower energy consumption (122 kJ/mol) at an estimated cost of $50–100 per ton CO_2_. This membrane-free design offers superior modularity and integration potential with offshore platforms (Figure 4). Direct seawater electrolysis presents the simplest underlying principle but remains largely theoretical, facing practical challenges such as electrode contamination and chloride side reactions [65].

**Figure 5:**
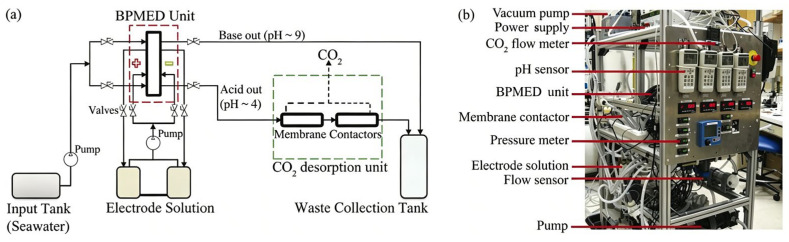
(**a**) Schematic of the BPMED experimental setup; (**b**) photo of experimental setup [36]. Reprinted with permission from [36]. Copyright 2012 Royal Society of Chemistry.

Currently, the bipolar membrane electrodialysis technique has emerged as the most extensively studied approach for electrochemical carbon capture methods [66]. This novel membrane separation technology demonstrates distinctive advantages, including: (1) configurable system architecture through modular design, (2) energy-efficient operation enabled by the low water dissociation voltage characteristic of bipolar membranes (BPMs), and (3) robust long-term operational stability under marine environmental conditions [67]. An early proposal by Eisaman et al. [36] to use the BPMED technique to separate DIC from seawater, and the schematic photo of the experimental setup, are shown in Figure 5. A nine-cell bipolar membrane electrodialysis (BPMED) system was built using polyethylene mesh spacers and EPDM sealing gaskets. Seawater and a 0.1 M H_2_SO_4_/0.25 M Na_2_SO_4_ electrolyte were pumped through different channels respectively. Under the electric field, H^+^ and OH^−^ ions migrated through selective ion-exchange membranes, thus forming acid/alkaline streams. The acidified solution was subjected to CO_2_ stripping by means of a membrane contactor under vacuum prior to being mixed with the alkaline effluent for discharge. Under steady-state conditions with constant flow rate and constant current, experimental results demonstrated DIC extraction efficiencies from seawater as CO_2_ of 33%, 60%, and 75% after one, two, and three passes through the membrane contactor, respectively, at the optimal flow rates of 3.75–5 lpm. For more practical applications, a flow rate of 6 lpm achieved an extraction efficiency of 68% with an energy consumption of 285 kJ·mol^−1^ (CO_2_) [36]. This innovative BPMED method provides a carbon recycling strategy through ion-exchange membrane-enhanced seawater carbon capture. However, issues like cell precipitation and low energy/decarbonization efficiency remain, establishing a foundation for future device optimization [68]. Chen et al. [69] further explored the efficiency of the optimized device and the mechanism of proton leakage in a continuous-feed percolation CA-BMED process, which provided important insights for the treatment of brine and proton leakage in CO_2_ capture through a continuous feed percolation by BPMED process.

Shen et al. [41] researched a transport-reaction differential model to predict the CO_2_ absorption–mineralization process in BPMED using seawater. This model accurately forecasts key performance indicators, including decalcification rate, carbon sequestration rate, and energy consumption, with its predictions validated across various conditions by experimental data. Digdaya et al. [70] optimized the BPMED system by integrating a pre-degassing membrane contactor to remove O_2_/N_2_ from seawater and replacing the electrolyte with K_3_/K_4_[Fe(CN)_6_] redox couples to reduce polarization losses, all within a modular design that enables scalable deployment without incurring additional reactions or voltage losses. Sharifian et al. [62] addressed CaCO_3_ precipitation in alkaline solution cells by optimizing electrode solutions, voltage, current density, and seawater flow rates through simulations and experiments. Their results indicated that the highest carbon capture efficiency was attained in the alkalinization solution cell at a pH between 9.6 and 10.0, where over 60% of DIC and more than 16% of Ca^2+^ could be removed. Additionally, it was essential to limit the current density to 5–10 mA cm^−2^ to achieve a total energy consumption of 318 kJ/mol, thereby maximizing the economic benefits in terms of electrical energy consumption and DIC removal efficiency [62]. Zhang et al. [71] developed a three-chamber porous electrolytic reactor for carbon capture, featuring a central chamber containing a Na_2_CO_3_/NaHCO_3_ solution, with the left and right chambers employing H_2_ electrolysis and water splitting, respectively. Protons (H^+^) generated in the left chamber migrate through a proton exchange membrane (PEM) to acidify the central solution, releasing high-purity CO_2_ gas [71]. Simultaneously, excess Na^+^ ions diffuse through a CEM into the right chamber, where they combine with OH^−^ produced by water electrolysis to regenerate NaOH for reuse. This system enables cyclic operation, achieving a CO_2_ capture rate of 964.5 mA cm^−2^, a Na^+^ transport efficiency exceeding 90%, and low energy consumption (118 kJ mol CO_2_^−1^ at 100 mA cm^−2^), while producing high-purity CO_2_ without gas mixing [71]. However, the high cost of custom electrodes and bipolar membranes limits their scalability and commercial application. Moreover, bipolar membranes pose a risk of toxic redox couple leakage into seawater, and the fouling generated during the reaction reduces the overall efficiency of the device. Furthermore, although field experiments of the BMED device have shown promising results, the specific impact of the membranes remains underexplored, and the scalability of the entire setup still requires further evaluation [72].

**Figure 6:**
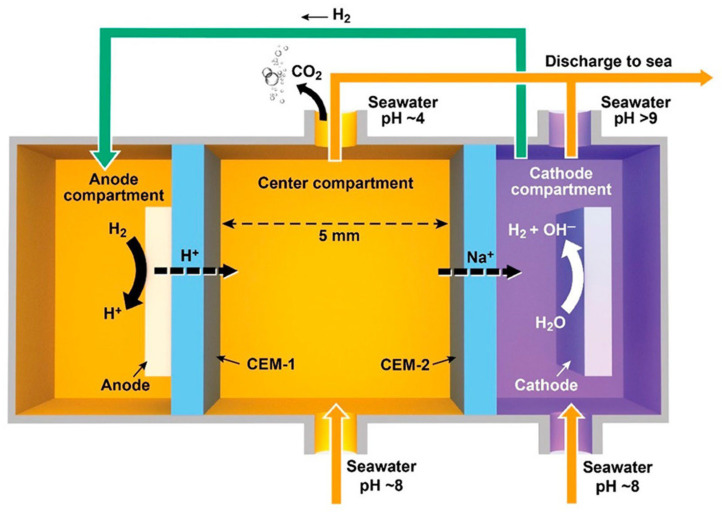
EHL cell schematic for CO_2_ removal from seawater [38]. Reprinted with permission from [38]. Copyright 2022 ACS Publications.

The EHL technology represents a distinct membrane-based electrochemical carbon capture approach, differing fundamentally from the BPMED [73]. The device employed in EHL technology is structurally divided into three primary cells—the left, middle, and right cells—each playing a critical role in preparing for the alkalinity-swing process, which is central to the system’s carbon capture mechanism [74]. Figure 6 illustrates a schematic diagram of the EHL cell designed for CO_2_ removal from seawater, where seawater electrolysis in the right cell generates OH^−^ and H_2_ [38]. The OH^−^ combines with Na^+^ through a cation exchange membrane to form an alkaline NaOH solution, while H_2_ transfers to the left cell and reacts at the anode to regenerate H^+^. These protons then migrate into the middle chamber, combining with Cl^−^ to produce an acidic HCl solution, which enables CO_2_ capture from acidified seawater via a membrane contactor.

Finally, both treated streams are returned to the ocean, utilizing electrolysis-induced micro-potentials to drive cation transport and achieve electrochemical hydrogen cycling [38]. The results show that the device saves about 33% of energy consumption compared with the BPMED technology and demonstrates the advantages of the technology in terms of high energy efficiency [38]. Furthermore, the EHL device eliminates the need for expensive components such as titanium plates, IrO_2_ catalysts, and electrode solutions required in the cathode/anode cells of BPMED. It only requires a small amount of hydrogen as the initial raw material for recycling, significantly reducing the overall material costs of the system [33]. One challenge associated with EHL technology is the potential adsorption of sodium, chloride, and other chemical ions on the anode during the transfer process, which can lead to reduced efficiency [75]. Additionally, Mg^2+^ or Ca^2+^ in seawater can react with hydroxides in the cathode cell to form Mg(OH)_2_ or Ca(OH)_2_ precipitates, necessitating a pretreatment step for seawater softening or precipitate removal [73]. Furthermore, periodic cleaning of the anode surface may be required to ensure long-term operational stability [74]. Despite the promising laboratory performance of BPMED and EHL systems, their scalability in corrosive marine environments faces fundamental challenges beyond those discussed above. Seawater contains not only Cl^−^ (which drives electrode corrosion) but also Mg^2+^, Ca^2+^, SO_4_^2−^, and diverse microorganisms—each interacting with electrochemical components in ways not replicated in synthetic seawater studies. BPMED systems require stable operation of bipolar membranes under variable salinity and temperature, yet membrane delamination and functional group loss accelerate in real seawater. EHL systems, while eliminating expensive ion-exchange membranes, depend on hydrogen recycling efficiency; any gas leakage or crossover reduces faradaic efficiency and creates safety hazards. Furthermore, both systems lack long-term validation (>1 year) in continuous-flow seawater conditions.

Additionally, an electrodialysis (ED) device developed by Prajapati et al. [76] resembles a BPMED system, featuring left, middle, and right chambers. In the left chamber, the anode electrolyzes water to generate H^+^, which acidifies the solution and facilitates CO_2_ release, while the cathode in the right chamber produces H_2_ and OH^−^, driven by a potential difference. This study concentrated on the role of the AEM in enhancing CO_2_ capture efficiency. The results demonstrated that, under optimal conditions (a 10 mm desalination chamber width and a flow rate of 10 mL/min), the AEM progressively favored the migration of HCO_3_^−^ (with similar selectivity for HSO_3_^−^) due to Cl^−^ accumulation in the acidic chamber, achieving a CO_2_ capture flux of 0.71 mmol/m^2^/s.

Based on the above review, we know that the ocean electrochemical carbon capture technologies offer a transformative solution for mitigating global carbon emissions, leveraging inherent advantages such as energy flexibility and modular scalability. Despite the transformative potential of electrochemical DOC, its path to commercialization is currently hindered by critical engineering constraints that compromise long-term operational reliability. Continuous exposure to the raw marine environment leads to electrode surface contamination and the potential adsorption of chemical ions, such as sodium and chloride, which significantly reduces current efficiency. This efficiency loss is further exacerbated by the competitive oxidation of Cl^−^ ions to Cl_2_ gas at the anode, a major side reaction that not only consumes additional energy but also necessitates complex gas management infrastructure. Simultaneously, the alkaline conditions required for CO_2_ mineral recovery or alkalinity enhancement trigger the precipitation of Mg(OH)_2_ and CaCO_3_. These mineral scales accumulate on membrane surfaces and within cell compartments, resulting in increased electrical resistance and shortened membrane lifetimes, which can lead to eventual operational failure without intensive seawater pretreatment or periodic polarity reversal strategies. Ultimately, these degradation and scaling mechanisms impose substantial long-term energy penalties, as voltage requirements must be increased over time to sustain target CO_2_ extraction rates, thereby directly undermining the technology’s overall economic competitiveness at scale.

## 6. Hollow Fiber Membrane Carbon Capture Technology Details

The development of membrane materials began in 2003 with Obuskovic et al.’s silicone oil-modified polypropylene hollow fiber membranes for VOC separation [77]. The essence of a hollow fiber membrane contactor is a highly efficient mass transfer device that combines the gas absorption process with membrane separation technology [78]. The core properties of membrane materials, including wetting resistance, liquid entry pressure, and pore structure stability, directly determine mass transfer efficiency and operational safety [79]. Its core driving force is the partial pressure difference of components on either side of the membrane, which constitutes the thermodynamic driving force for mass transfer [50]. In this process, CO_2_ in the gas phase first diffuses through the membrane pores and then reacts with or physically dissolves into the flowing absorbent at the membrane–liquid interface. This process follows the “resistance-in-series” model, where the total mass transfer resistance is jointly determined by the gas-phase boundary layer resistance, the membrane resistance itself, and the liquid-phase boundary layer resistance [80]. The structure and surface properties of the membrane are crucial for maintaining a stable gas–liquid interface, preventing liquid penetration, and thus ensuring high-efficiency mass transfer [50].

In recent years, significant progress has been made in CO_2_ capture applications. Notably, hollow fiber membrane contactors traditionally used for flue gas treatment have been successfully adapted for direct air capture (DAC) through the following key modifications: (1) adoption of high-selectivity membrane materials as reviewed by Shiravi [50] to enhance capture efficiency for low-concentration CO_2_ (~400 ppm); (2) incorporation of AgNPs/GO composite membrane technology developed by Zhang et al. [81] to address membrane fouling caused by atmospheric dust; and (3) integration of Olabi’s [82] spiral flow channel design to optimize mass transfer efficiency under low air flow velocities. Two key application scenarios of this technology must be clearly distinguished with DAC and DOC. DAC refers to direct CO_2_ capture from ambient air or flue gas, while DOC specifically targets CO_2_ extraction from seawater or marine-related aqueous solutions, which is the focus of this study. For DOC applications, membranes must possess excellent fouling resistance, chemical stability in seawater, and resistance to salt precipitation and scaling. Mansourizadeh’s [79,83] porous PVDF membranes and Fashandi’s [84] optimized PVC membranes further improved system stability under varying humidity conditions, enabling membrane contactors to maintain >90% CO_2_ capture efficiency in DAC applications. These material modifications simultaneously enhance wetting resistance and pore structure stability, ensuring stable long-term operational performance. These adaptive modifications provide new pathways for developing high-efficiency, low-energy direct air capture technologies. However, such efficiency highly depends on operating conditions (e.g., flow rate, CO_2_ partial pressure, temperature), and has limitations such as membrane wetting and performance degradation due to dominant gas-phase resistance at extremely low CO_2_ concentrations.

In addition, Chen et al. [85] conducted a theoretical study on CO_2_ extraction from acidic seawater using finite-time thermodynamics, deriving analytical expressions for CO_2_ extraction efficiency and the entropy generation rate (EGR), as well as investigating the related optimization strategies. According to the experiments, the extraction rate of CO_2_ can be more than 98%, and the effects of various factors on the extraction rate and EGR are elucidated, which provide theoretical guidance for ocean carbon capture [85].

**Figure 7:**
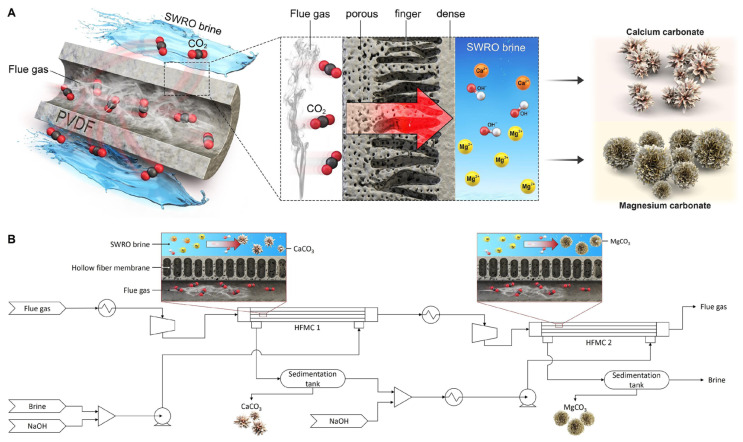
Illustration of the direct CO_2_ mineralization system: (**A**) Schematic diagram of the hollow fiber membrane contactor (HFMC) system utilizing Ca^2+^ and Mg^2+^ in seawater reverse osmosis (SWRO) brine for CO_2_ mineralization; (**B**) Process flow diagram of a series-connected HFMC system designed for selective carbonation of Ca^2+^ and Mg^2+^ [86]. Reprinted with permission from [86]. Copyright 2024 Elsevier.

**Figure 8:**
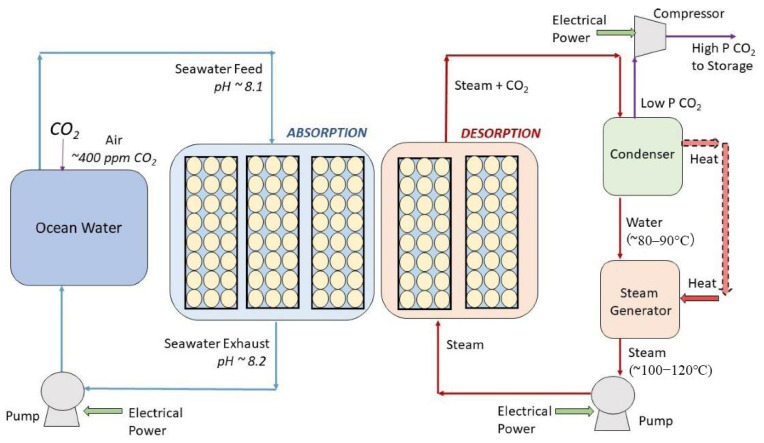
A direct ocean capture system utilizing encapsulated solvents operates by flowing seawater through a parallel-arranged capsule bed (yellow circle in column), where the capsules absorb carbon dioxide from the seawater [88]. Reprinted with permission from [88]. Copyright 2023 Elsevier.

Lee et al. [86] achieved the direct mineralization of carbon dioxide from seawater reverse osmosis brine with the help of a hollow fiber membrane contactor (Figure 7). The device was specifically designed to overcome the gas–liquid–solid three-phase contact issues typically found in conventional mineralization units. In high-salinity seawater environments, membrane fouling resistance, chemical stability, and resistance to salt precipitation and scaling are critical for stable operation. The characteristics of the membrane improved the mass transfer efficiency of CO_2_, resulting in a 94% reduction in CO_2_ removal. The properties of the membrane enhanced the mass transfer efficiency of carbon dioxide [86]. Furthermore, the techno-economic analysis highlights the advantages of this system over conventional amine scrubbing, which enables a 35% lower cost of CO_2_ capture with valuable product, providing a promising solution for carbon capture [86]. While the laboratory results are promising, several factors (membrane area requirements, capital and operating cost estimates, membrane lifetime, module packing, and footprint and biofouling management) must be assessed before claiming industrial feasibility for DOC applications. In subsequent research on various HF membranes, it was found that a highly porous (three-layer) membrane structure is more conducive to improving the carbon dioxide capture efficiency. The mechanisms by which the gas and liquid flow rates affect the capture rate were clarified, and the importance of membrane surface modification and system parameter optimization was emphasized, providing theoretical and experimental support for the field of CO_2_ mineralization [87]. Austin et al. conducted a study of direct carbon capture from seawater using microencapsulated solvent technology (Figure 8), which provides a large membrane specific surface area for CO_2_ capture, and the contained solution can be reused [88]. In addition, this study performed lab-scale operations with one-dimensional modeling and techno-economic analysis to support the feasibility of this solution [88].

Overall, hollow fiber membrane technology shows great potential in the field of carbon capture. However, to achieve large-scale industrial application, continuous exploration and innovation are still needed in aspects such as membrane material research and development, membrane module design, and process optimization. With the continuous development of materials science, manufacturing processes, and related technologies, the hollow fiber membrane carbon capture technology is expected to become an important means to address climate change and achieve carbon emission reduction goals [82,89].

## 7. Discussion and Perspectives

### 7.1. Discussion

**Figure 9:**
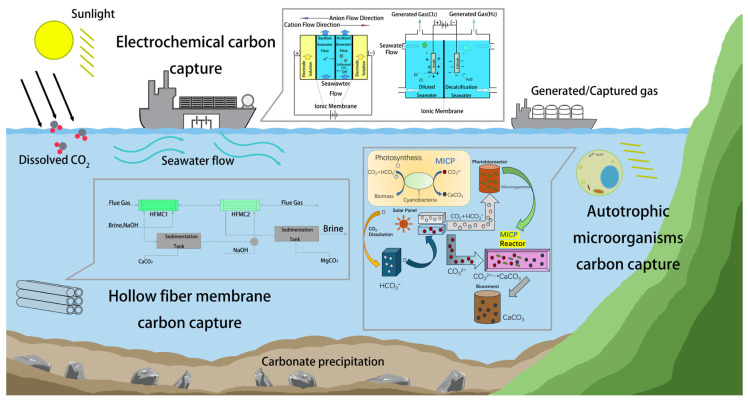
Integrated development roadmap of biological, electrochemical, and hollow fiber membrane-based DOC systems.

Across the three DOC technology categories, several common evaluation gaps emerge from the literature. Comparative advantages are rarely quantified using consistent metrics, as most studies report performance under unique experimental conditions. Scalability constraints such as membrane area requirements and electrode lifetime are often acknowledged but rarely quantified. Environmental risks including alkalinity shifts and biofouling remain largely speculative, with no continuous monitoring reported beyond laboratory mesocosms. Technology readiness levels (TRL 4–6) are based on laboratory demonstrations. In addition, reported efficiencies represent optimal laboratory conditions; real-world factors could reduce performance by 30–50% in continuous marine operation. The comparative techno-economic analysis reveals distinct trade-offs among the three DOC technologies. Electrochemical methods achieve the highest DIC removal efficiency with moderate energy consumption and costs, but laboratory-optimized metrics assume ideal conditions. Membrane-based methods offer high removal efficiency, but these results were achieved at CO_2_ concentrations much higher than those in open seawater. At commercial scale, membrane scaling, electrode degradation, and biofouling are expected to significantly increase energy consumption and reduce membrane lifetime in seawater. Biological methods have the lowest energy input (solar-driven) but slow growth kinetics and a large land footprint. On a levelized cost basis accounting for scalability constraints, electrochemical methods currently offer the most promising pathway, provided that electrode fouling and stability challenges are resolved through continued materials research. To visually synthesize the comparative analysis and future outlook presented above, Figure 9 presents an integrated development roadmap of biological, electrochemical, and hollow fiber membrane-based direct ocean carbon capture systems.

Compared to DAC or post-combustion carbon capture (PCC), DOC technology remains relatively underdeveloped [90]. The comparative analysis of PCC, DAC, and DOC technologies reveals critical insights into their respective roles in global carbon management strategies. While PCC currently dominates operational deployment due to its technological maturity and lower costs ($40–80/ton CO_2_) [91], its long-term potential is constrained by dependence on fossil fuel infrastructure. In contrast, DAC and DOC offer more flexible carbon removal solutions but face significant challenges in energy efficiency and scalability [92].

**Figure 10:**
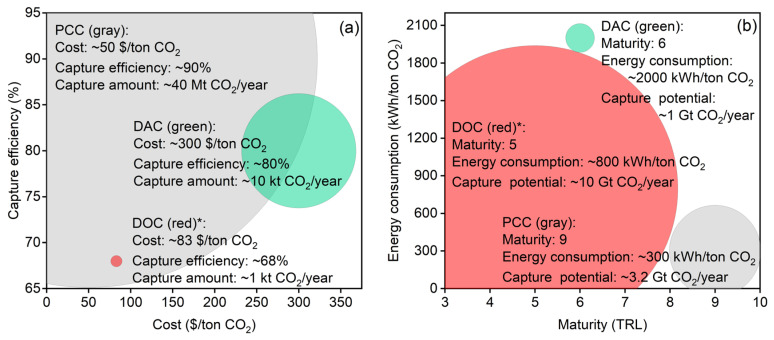
(**a**) Economic comparison of three carbon capture technologies of PCC, DAC, and DOC in bubble plots, where the bubble size represents the annual amount of CO_2_ captured by the technology; (**b**) comparison of energy consumption and scale potential for PCC, DAC, and DOC technologies, where the bubble size represents the annual CO_2_ capture potential of the technology. The data presented in this figure are derived from references or reports [36,64,91,93,94,96,97,98,99,100,101,102,103]. DOC (red)*: represents the focus of this study.

Figure 10a presents a comprehensive techno-economic comparison of three carbon capture technologies using a bubble chart visualization, where bubble size corresponds to annual capture capacity. PCC demonstrates superior cost–efficiency balance (40–80 USD/ton at 85–95% efficiency), though limited to point sources [93]. DOC shows intermediate performance (50–150 USD/ton, 60–85% efficiency) with a smaller deployment scale [36,94], while DAC exhibits the highest costs (250–600 USD/ton) despite good efficiency (70–90%) [91]. Figure 10b shows a comparison of energy consumption and scale potential for PCC, DAC, and DOC, where the bubble size corresponds to the annual CO_2_ capture potential. Here, PCC shows full commercialization (TRL 8–9) with established cost structures but faces geographical constraints [91]. DAC (TRL 6–7) benefits from strong policy incentives but suffers from cost uncertainties at scale [95]. DOC remains in the demonstration phase (TRL 4–5), with significant marine deployment potential, pending environmental impact studies. Critically, these comparisons have important limitations. First, the cost and efficiency data are drawn from studies with different system boundaries, assumptions, and experimental conditions, making direct comparison inherently uncertain. Second, laboratory-scale efficiencies for DOC (60–85%) have not been validated at pilot or commercial scale; performance decay due to fouling, scaling, and degradation is not yet quantified. Third, DAC costs remain highly uncertain, with recent estimates varying by a factor of two depending on energy source and capture technology. Fourth, the comparison does not account for regional factors such as energy prices, regulatory frameworks, or disposal costs. Therefore, while Figure 10 provides a useful high-level comparison, these data should be interpreted as indicative ranges rather than absolute performance guarantees, and pilot-scale validation under real-world conditions remains essential.

These data demonstrate that, while PCC currently dominates industrial applications, emerging DOC technologies could provide critical marine-based solutions if current challenges in energy efficiency and ecological impacts are addressed. Given these current technological limitations, DOC may reach pilot-scale validation (10–100 tons CO_2_/year) by 2030, with commercial viability potentially achievable by 2035–2040, provided that current material and process challenges—particularly membrane fouling, electrode degradation, and biofouling—are resolved through sustained research efforts. Future research should prioritize life-cycle assessments and pilot-scale demonstrations to validate marine carbon removal potential while minimizing ecosystem disturbances. Such technological advances, coupled with standardized carbon accounting frameworks, could position DOC as a sustainable complement to existing land-based capture methods in global decarbonization strategies.

### 7.2. Marine Environmental Implications of DOC Technologies

Despite its promising carbon removal potential, large-scale implementation of DOC could significantly alter local seawater chemistry, including changes in alkalinity, pH, and carbonate saturation states [104]. Such modifications may disrupt the natural buffering system of seawater, increase risks of regional acidification, and threaten calcifying organisms such as bacteria, sea grass, and fish [105]. In addition, frequent adjustments of solution chemistry during DOC operation can accelerate precipitation of secondary minerals such as calcium carbonate and magnesium carbonate, which may accumulate on membranes and seafloor sediments, further disturbing benthic ecosystems and habitat stability [86,87]. Furthermore, concentrated brine generated during DOC processes exhibits higher salinity and altered ionic composition; its direct discharge can change local osmotic pressure, damage marine organisms, and reduce coastal biodiversity [106]. Meanwhile, biofouling and chemical cleaning agents may introduce additional chemical stressors to the marine environment, highlighting the urgent need for standardized ecological risk assessment and long-term monitoring protocols [107]. In summary, current understanding of these environmental implications is based largely on laboratory observations and modeling. Pilot-scale environmental monitoring studies are urgently needed to validate these potential impacts before commercial-scale DOC deployment proceeds. A systematic comparison of key performance indicators for the three DOC technology categories is presented in Table 1, covering efficiency, energy, cost, TRL, scalability, environmental footprint, deployment scenarios, risks, and durability.

## 8. Conclusions

In conclusion, direct ocean carbon capture (DOC) represents an emerging framework for atmospheric CO_2_ mitigation, though current progress remains primarily confined to laboratory and pilot-scale demonstrations (TRL 4–5), requiring a cautious assessment of its industrial viability. Biological strategies, including microalgae-based capture and diatom/MnOx systems, demonstrate photosynthetic efficiencies between 10% and 20%, yet their practical adoption is constrained by slow capture kinetics, high nutrient dependency, and breakeven costs that can reach $920 per ton of CO_2_. Electrochemical approaches, particularly BPMED, provide high capture intensities but face significant engineering challenges such as electrode corrosion, competitive side reactions like chlorine evolution, and high energy demands ranging from 118 to 730 kJ/mol. Furthermore, while hollow fiber membrane technology offers superior mass transfer characteristics, its long-term operational stability is frequently compromised by biofouling and membrane wetting under harsh marine conditions, which drastically increases mass transfer resistance. Consequently, although the integration of bio-promotive materials into hybrid systems offers a theoretical route for scalable carbon sequestration, the transition to commercial implementation depends on rigorous pilot-scale validation and comprehensive life-cycle assessments to ensure both economic competitiveness and the minimization of unpredictable ecological disturbances. To accelerate the transition of DOC from demonstration to commercial viability, future research should prioritize the following strategic areas: focused research should prioritize anti-fouling membranes with biomimetic surfaces and long-term field validation, alongside low-energy electrochemical pH swing systems targeting <50 kJ/mol CO_2_ through chloride-tolerant electrodes and waste heat integration; hybrid DOC-DAC configurations offer synergistic benefits by combining bulk ocean capture with air polishing; offshore modular integration requires compact reactors powered by renewable energy with on-board CO_2_ utilization; pilot-scale demonstrations (10–100 tons CO_2_/year) with continuous environmental monitoring are urgently needed to validate performance and assess ecological impacts.

## Figures and Tables

**Table 1:** Systematic comparison of key performance indicators for biological, electrochemical, and membrane-based DOC technologies.

Performance Indicator	Biological [31,56]	Electrochemical [20,33,36,38,62,64,74]	Membrane-Based [21,50,86,88]
CO_2_ capture efficiency	10–20% (photosynthetic efficiency)	60–87% (DIC removal)	70–94% (lab scale, optimized conditions)
Energy consumption	Low (solar-driven); 0.5–1.5 kWh/kg biomass for harvesting	80–500 kJ/mol CO_2_ (EHL: ~80; BPMED: 300–500)	0.5–2 kWh/m^3^ seawater for pumping; vacuum for stripping
Cost estimates (USD/ton CO_2_)	$230–920	$50–150	$100–200
TRL	5–6 (pilot-scale demonstrated)	4–6 (BPMED: 5–6; EHL: 4–5)	4–5 (lab to pilot; no commercial deployment)
Scalability	Low–moderate (land-intensive, slow kinetics)	High (modular design, stackable cells)	Moderate (module-based, but fouling limits long-term operation)
Durability	Limited by culture stability and contamination risk	Electrode degradation in Cl^−^ -rich environment; membrane scaling	Membrane wetting (30–70% flux reduction); biofouling
Environmental footprint	Large land and water footprint; nutrient discharge	Low–moderate (brine discharge; mineral sludge)	Low–moderate (chemical cleaning waste; membrane disposal)
Deployment scenarios	Offshore standalone renewable-powered systems; bioplastic monomer production	Onshore or offshore (modular); mobile possible	Onshore preferred; offshore emerging
Risks	Long-term field stability and large-scale applicability require further validation; reactor biofouling risk; nutrient discharge	Metal release caused by dissolution of silver electrode; Cl_2_ emission; membrane scaling	Membrane wetting; biofouling; performance decay

## Data Availability

No new data were created or analyzed in this study. Data sharing is not applicable to this article.

## Abbreviations

The following abbreviations are used in this manuscript:

AEM	anion exchange membrane
BPMED	bipolar membrane electrodialysis
CCS	carbon capture and storage
CEM	cation exchange membrane
CO_2_	carbon dioxide
DAC	direct air carbon capture
DIC	dissolved inorganic carbon
DOC	direct ocean carbon capture
EHL	electrochemical hydrogen-looping
IPCC	Intergovernmental Panel on Climate Change
MICP	microbially induced carbonate precipitation
Mn	manganese
MNBs	micro-nano-bubbles
MnOx	manganese oxides
NPP	net primary productivity
PCC	post-combustion carbon capture
POC	particulate organic carbon
TPP	total primary productivity
XAS	X-ray absorption spectroscopy
